# Classification of marine microdebris: A review and case study on fish from the Great Barrier Reef, Australia

**DOI:** 10.1038/s41598-018-34590-6

**Published:** 2018-11-06

**Authors:** Frederieke J. Kroon, Cherie E. Motti, Lene H. Jensen, Kathryn L. E. Berry

**Affiliations:** Australian Institute of Marine Science (AIMS), Townsville, Qld 4810 Australia

## Abstract

Marine debris, and in particular plastic pollution, is ubiquitous throughout global marine environments. Here, we present a classification of marine microdebris (i.e. debris between 0.1 μm and <5 mm) tailored to represent synthetic, semi-synthetic and naturally-derived items. The specific aim of this classification is to introduce a level of consistency in the higher-level characterisation of marine microdebris, thereby improving the overall reporting on marine microdebris contamination. We first conducted an extensive literature review on the accumulation of ingested debris in fish to identify discrepancies in marine microdebris reporting as a basis for the new classification. The review reveals the diverse nature of ingested marine microdebris, including items that are non-plastic but often incorrectly reported on as microplastics. We then applied our classification to a case study on wild-caught juvenile coral trout, *Plectropomus* spp., from the Great Barrier Reef World Heritage Area, Australia. This first report on accumulation of ingested marine debris in commercial fish on the reef demonstrates a high frequency of occurrence and a prevalence of semi-synthetic and naturally-derived fibres. Based on our findings, we offer recommendations on potential improvements for the classification presented, ultimately contributing to a more realistic assessment of the ecological risks of marine microdebris.

## Introduction

Marine debris is defined as ‘*any persistent, manufactured or processed solid material discarded, disposed of or abandoned in the marine and coastal environment*’^1^. These materials have been manufactured, modified or used by people, with plastics generally constituting the most common items of marine debris^1,2^. Recent reviews have found that marine debris is pervasive and has been documented in marine habitats, organisms and ecosystems worldwide^3^. Marine debris can be categorized according to size^4^, and is defined for plastics as mega (>1 m diameter), macro (between 2.5 cm and <1 m), meso (between 5 mm and <2.5 cm), micro (between 0.1 μm and <5 mm) and nano (<0.1 μm)^5^. Such categorisation of marine debris greatly improves our ability to compare contamination across studies, and can contribute to determining the sources, transport and fate in the marine environment^3,6^.

Following the seminal paper on marine microplastic contamination by Thompson *et al*.^7^, this particular type of marine debris has received increasing attention^8^. Indeed, the presence of microplastics in marine environments has been identified as an emerging issue of international concern^5^. Microplastic contamination has been reported for coastlines^7^, sub-surface waters^9^, water columns^10^, benthic sediments^11^, and deep sea floors^12^. Accumulation of ingested microplastics has been documented in a large number of wild caught organisms^13^ ranging from zooplankton^14^ to marine megafauna^15^, including species for human consumption^3^. Assessing the exposure of, and potential effects on marine organisms to microplastics, however, is complicated due to the large variety of polymer types, as well as sizes, shapes, and other characteristics^16,17^. This is further exacerbated by the fact that non-plastic microdebris sometimes is misidentified and incorrectly reported as microplastics^18,19^, even though they do not contain any synthetic polymers.

To determine the impact of marine microdebris, and of microplastics in particular, the use of standardised procedures and terminology for accurate characterisation and description has become increasingly important^16,17^. This is elucidated by recent reports that non-plastic items can constitute a considerable proportion of the total amount of observed marine microdebris in abiotic environments^20,21^ and biotic samples^18,19^. To accurately characterise the chemical composition of marine microdebris, including removing the possibility of false positives for microplastics^22^, the use of spectroscopy is crucial^16,23^. Standardised application of analysis workflows developed for spectroscopy^20^ will further reduce bias in chemical type identification and improve confidence in contamination estimates. In contrast, the use of standardised terminology to describe marine microdebris based on chemical type has received relatively little attention. Recent studies have classified marine microdebris into synthetic and semi-synthetic items^18,21,24^, but did not provide clear definitions for these classifications. Hence, the specific aim of this study is to present a classification system thereby introducing a level of consistency in the higher-level characterization of marine microdebris. The application of such a classification system will provide improved certainty to distribution and abundance data of different chemical types, and will ultimately contribute to a more realistic assessment of potential ecological risks of marine microdebris.

In this study, we firstly conduct a comprehensive literature review on ingestion of marine debris in coastal, marine and oceanic fish to identify discrepancies between (i) the classification and characterisation of, and (ii) the reporting of metrics on ingested marine debris. These discrepancies and potential mis-classifications were used as guidelines to develop a new classification system tailored to represent synthetic, as well as semi-synthetic and naturally-derived items. We then applied this classification system to examine the accumulation of ingested marine debris in wild-caught juvenile coral trout, *Plectropomus* spp. (Family Serranidae), from the Great Barrier Reef (GBR) World Heritage Area (WHA), Australia. Coral trout from the genus *Plectropomus* are large piscivorous teleosts and constitute most of the commercial and recreational fisheries catch in the GBR WHA^25^. Potential marine debris items (both particles and fibres), separated visually from coral trout gastrointestinal tract (GIT) using stereomicroscopy, were subsequently physically characterised using microscopic photography, and chemically characterised using attenuated total reflectance (ATR) Fourier transform infrared (FTIR) spectroscopy, following the workflow outlined in Kroon *et al*.^20^. Based on these results, and in combination with the findings from the literature review, we offer recommendations on potential improvements for the characterisation and classification of marine microdebris.

## Results

### Marine debris ingestion in coastal, marine and oceanic fish

A total of 66 studies on marine debris ingestion by coastal, marine and oceanic fish, published in the primary scientific and grey literature from 1970 to 2017 were reviewed (Supplementary Table 1). These studies examined at least (i) 343 different species with others undescribed to species level (e.g. *Lampris* spp.^26^; unidentified fish larvae^14^), and (ii) 25,083 individual fish although the exact numbers of fish examined was not always available and is likely to be much higher (e.g. >500 fish in Colton *et al*.^27^; >3,000 fish in Govoni, pers. comm.^28^).

To identify and characterise the accumulation of ingested marine debris in fish, these studies used visual separation, chemical separation, photography and spectroscopy, either in isolation or in combination (Supplementary Table 1). The ingestion of synthetic debris, namely pieces of plastic and rubber, was first documented in the lancetfish *Lepisaurus ferox* in Japan in 1970^29^. The accumulation of ingested microplastics by fish, visually identified as polystyrene spherules, was first reported for seven different fish species in Niantic Bay, U.S.A. in 1972^30^. The authors classified these ingested spherules (0.1–2 mm diameter) as plastic based on physical similarities to spherules found in associated waters and confirmed to be polystyrene by infrared spectroscopy. Other studies have also inferred the synthetic origin of ingested microdebris from visual examination^31,32^. However, recent work has demonstrated that spectroscopy is essential to confirm a synthetic origin for particles <2.0 mm^20^ and fibres of any size^23^. Spectroscopy was first applied on marine debris ingested by fish in 2013^18,33^. Foekema *et al*.^33^ demonstrated the synthetic origin of a sub-set of particles separated from North Sea fish, namely polyethylene, polypropylene, polyethylene terephthalate and styrene acrylate. Synthetic particles and fibres were also detected in fish by Lusher *et al*.^18^ and since by many other studies using spectroscopy (Supplementary Table S1).

The accumulation of ingested semi-synthetic marine debris, specifically fibres, was first reported for pelagic and demersal fish from the English Channel in 2013^18^ (Supplementary Table S1). In this study, semi-synthetic fibres made of the cellulosic material rayon were the most common microdebris items (58%) detected. Indeed, when microfibers are considered in fish ingestion studies, they can make up a significant proportion of the ingested items (e.g. ≥80%)^34–37^. Such fibres are often classified as plastic following visual inspection^38,39^, however, putative synthetic microfibres have been shown by spectroscopy to be semi-synthetic or naturally-derived^19,23,40^. The prevalence of semi-synthetic fibres, including rayon^18,19,41,42^ and cellophane^35,43,44^, was confirmed using spectroscopy in recent fish ingestion studies.

Recent spectroscopy studies have also identified marine debris of natural origin, including cellulose^40^, cotton and wool^43^ fibres, and keratin and chitin^44,45^ particles (Supplementary Table S1). None of these studies subsequently examined the physical characteristics of these items to establish whether they had been modified or used by people. For fibres these include properties like equal thickness, no tapering towards the ends, a three-dimensional bending (i.e. not entirely straight), a clear or homogenous colouration, as well as the presence of a yarn or intertwined structure^16,46^. For particles, these include shape (e.g. equal thickness), texture (e.g. patterned), and colour presence and homogeneity (e.g. blue, red, black, yellow)^16,46^. Such distinction is important as items from human usage (i.e. naturally-derived) would normally not have entered the marine environment, and could have physical and chemical characteristics that may affect marine organisms and ecosystems.

The reporting of metrics on ingested marine debris in fish is highly variable; consequently cross-study comparisons are fraught with difficulties (Supplementary Table 1). First, studies do not always specify which part of the GIT was examined^47^, or only examine one component of the GIT^35,44^. To ensure comparability within and across studies, the presence and abundance of ingested debris should be reported based on the specific section of the GIT examined (e.g. stomach, intestines and rectum)^44^. Second, methods to separate ingested marine debris from gut contents range from visual examination of macro- and mesodebris in earlier studies^29,30^ and microdebris in more recent studies^18^, to chemical digestion of GIT^33^ and whole fish^48^ (Supplementary Table S1). These different methods are likely to affect estimates of ingested marine debris, as chemical type cannot accurately be confirmed using visual examination only^20,23^, and chemical digestion can affect recovery of marine microdebris^49^. Third, not all studies report on the number of individual fish per species that have ingested marine debris or the frequency of occurrence of marine debris ingestion per species^37,44,50^. For those that do, zero was reported most often for individual fish (n = 157, 44%) for a total of 357 taxa, and for frequency of occurrence (n = 112, 36%) for a total of 309 taxa. Across the 66 studies, 3,989 (16%) of the 25,083 individual fish examined were found to have ingested marine debris. The average number of marine debris items per fish species is also not always reported^44,50^, or is only reported as an estimate based on fish that have ingested marine debris^18,51^. Across those studies that did consider all individual fish examined per species, the highest average and maximum number of marine debris items recorded is 7.2 ± 8.4 standard deviation (s.d.) for *Symbolophorus californiensis* and 83 for all species examined in the North Pacific Central Gyre^52^. Importantly, non-synthetic marine microdebris items are often included as microplastics in reporting metrics^18,19^, resulting in unrealistic estimates of microplastics ingestion by coastal, marine and oceanic fish.

### Definitions for classification

Based on the findings from our literature review, we present and apply the following classification of marine microdebris to examine the accumulation of ingested marine debris in wild-caught juvenile coral trout, *Plectropomus* spp.:

- **Synthetic**: items manufactured by chemical synthesis, including through the process of polymerization. This includes thermoplastics (e.g. nylon, polyethylene, polypropylene, and polystyrene), and thermoset and elastomer plastics (e.g. polyester, polysiloxane, and polyurethane).
- **Semi-synthetic**: items manufactured synthetically from one or more substances of natural origin. This includes materials regenerated from one or more natural substances (e.g. rayon derived from cellulose), and materials that are composites of natural and synthetic substances (i.e. natural fibre reinforced polymer composites, NFPC)^53^.
- **Naturally-derived**: items manufactured from one or more substance of natural origin. This includes materials derived from plants (e.g. cotton, flax, hemp, linen, ramie) and animals (e.g. wool, fur), and from inorganics (e.g. calcium carbonate, calcium silicate). Materials that are composites of two or more natural substances (e.g. mixed yarns from natural fibres) are also included here.

### Processing and analyses of marine debris in juvenile coral trout

The accumulation of ingested marine debris was examined in 20 juvenile coral trout (*Plectropomus* spp) collected around four geographically separated reef islands along the length of the GBR WHA in 2011 (Table 1).The number of potential marine debris items included and excluded from the analyses are presented for each step of the workflow applied to the 20 GIT samples (Table 2). Potential marine debris items (n = 200), comprising both fibres (n = 164, 82%) and particles (n = 36, 18%), were detected in all 20 juvenile coral trout following visual examination under a stereomicroscope. A total of 189 individual items (156 fibres, 33 particles) were analysed by ATR-FTIR spectroscopy. The discrepancy between the number of items identified (200) and analysed (189) was due to items missing from their slide (n = 5) or being too small for ATR-FTIR analysis (n = 6). Of the 189 individual items, 166 (88%) matched ≥70% to spectra in the Nicodom IR libraries, while 11 (6%) showed a lesser match of between 60 and <70%. Twelve (6%) items had a match of <60% and were not considered for further analyses^18,20^. Key diagnostic signals were not detected for five of the 11 items with intermediate matches to library spectra (between 60 and <70%), likely due to co-occurrence with other components. Comparative analyses of the spectra of these five items against all 189 spectra resulted primarily in matches with other particles that also had matches between 60 and <70%. Given the low confidence in their ATR-FTIR spectra and a lack of diagnostic chemical signals, these five items were subsequently excluded from further analyses. Hence, of the 189 items initially analysed by ATR-FTIR, a total of 17 (12 + 5) were eliminated due to poor matches to the Nicodom IR libraries and the lack of diagnostic chemical signals in their spectra. A total of 172 individual items (145 fibres, 27 particles) were included in further analyses (Table 2).

### Preventing contamination of juvenile coral trout samples

During the dissection and processing of the samples in the AIMS laboratory, 33 fibres and six particles were collected in the four petri dishes placed adjacent to the work area (i.e. procedural blank controls). In addition, reference samples of materials used during collection, dissection, and processing (n = 7) were retained. The structure, shape, texture and colour of these items were noted, their chemical composition analysed by ATR-FTIR spectroscopy as described above, and their spectra added to the customised contamination library. Spectral comparison of the 172 potential marine debris items with those in the customised contaminant library revealed that 71 items showed a ≥90% chemical correlation to one or more known contaminants (Table 2). These matches were primarily to spectra from fibres collected in the petri dishes. Visual inspection and comparison of the physical characteristics of these 71 items with those used to build the customised contaminant library, found that for 19 there were strong similarities in both spectra (i.e. ≥90% match to the contaminant) and physical characteristics (i.e. size, shape, texture, and colour). As these 19 items could not be excluded as potential contaminants in the samples they were therefore not considered in any further analyses (Table 2).

### Chemical characterisation of marine debris in juvenile coral trout

Based on ATR-FTIR and the subsequent spectral interrogation (search and compare analyses), the chemical composition of 70 (46%) of the remaining 153 potential marine debris items (126 fibres, 27 particles) indicated they were potentially of natural origin. These 70 included 37 keratin-derived fibres and particles, and 26 cotton (cellulose) fibres. Closer inspection of their physical characteristics revealed that a total of 38 items, namely (i) two aragonite particles, (ii) 12 keratin fibres and 19 keratin particles, (iii) two cotton fibres, (iv) one pigment particle, and (v) two silicate particles, were likely of natural origin (Supplementary Text 1). Thus, a final total of 115 (75%) out of the 153 items were assigned to be marine debris (Table 2), comprising 58% of the 200 potential marine debris items that were first visually separated using stereomicroscopy.

**Figure 1:**
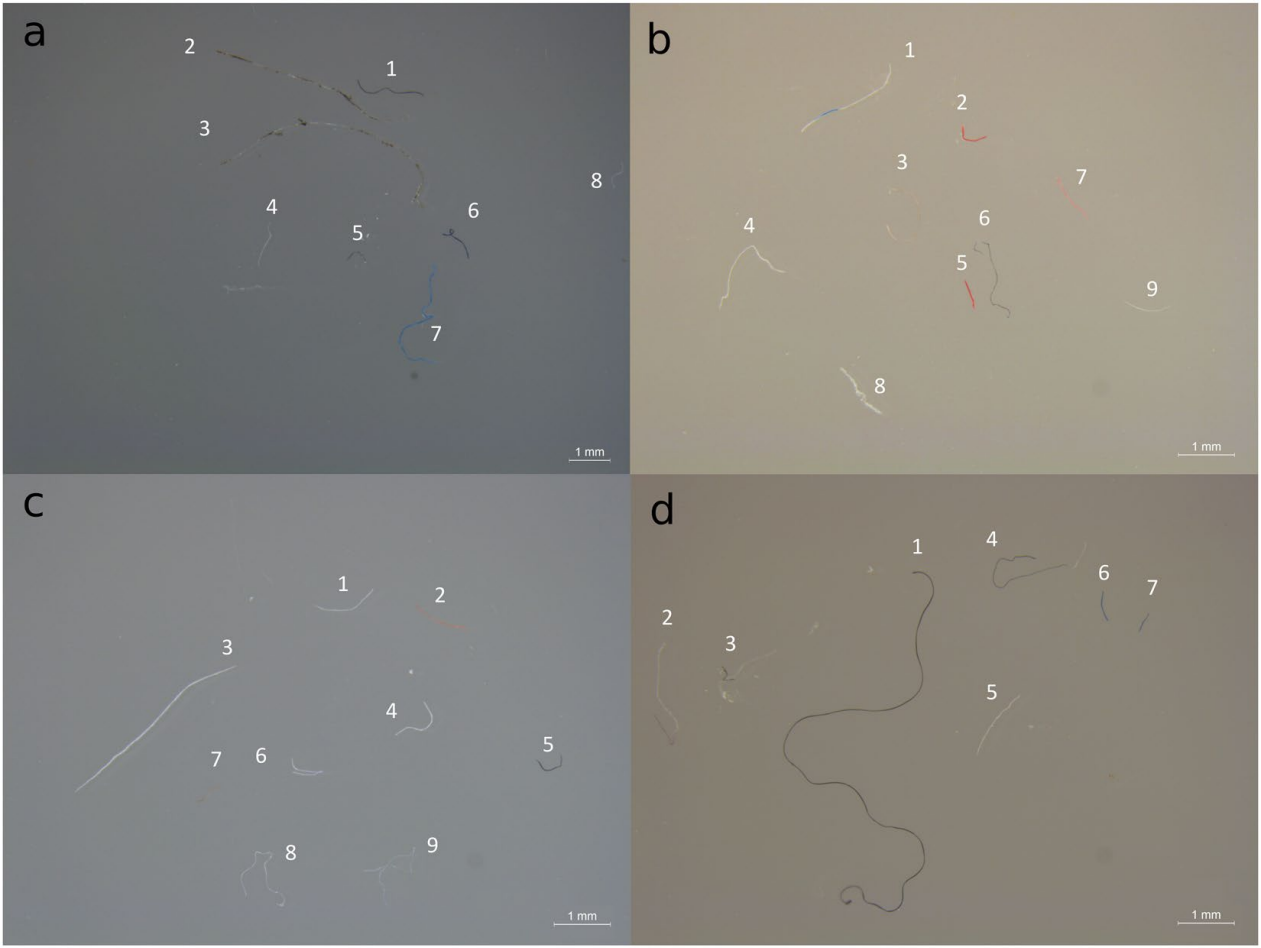
Photographs of representative marine microdebris in juvenile coral trout. Marine microdebris was detected in the gastrointestinal tract of 20 individual juvenile coral trout (*Plectropomus leopardus* and *P. maculatus*) collected on reefs around (**a**) Lizard Island, (**b**) Orpheus Island, (**c**) Heron Island, and (**d**) One Tree Island, in the Great Barrier Reef World Heritage Area, Australia, in 2011. Examples of chemical type assignments for marine microdebris include: (**a**) *semi-synthetic*: cellulose-derived (4), NFPC: nylon (5); *naturally-derived*: cellulose-natural (1, 6, 7); (**b**) *semi-synthetic*: cellulose-derived (1, 2, 3, 5, 6), NFPC: nylon (8); *naturally-derived*: cellulose-natural (7), keratin (4); (**c**) *synthetic*: polyester (8); *semi-synthetic*: NFPC: nylon (5, 6), NFPC: polyester (1); *naturally-derived*: cellulose-natural (2, 3, 9); (**d**) *semi-synthetic*: cellulose-derived (6), NFPC: nylon (5), NFPC: polyurethane (1); *naturally-derived*: cellulose-natural (4, 7), keratin (3). For the following items, natural origin (a2, a3, a8) or contamination (b9, c4) could not be ruled out. Chemical type was not assigned to fibre c7 (fibre was missing) and d2 (<60% match). NFPC = natural fibre reinforced polymer composites.

**Figure 2:**
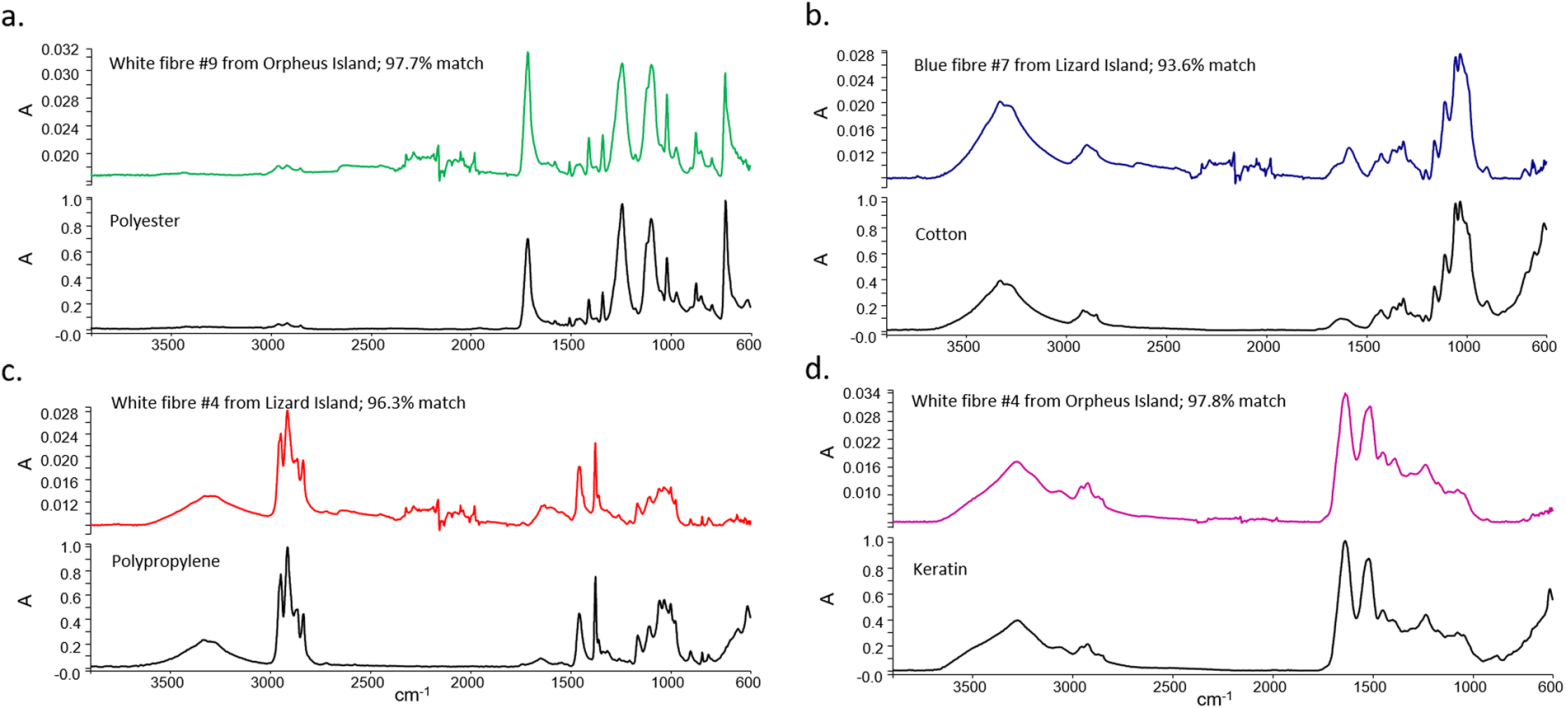
ATR-FTIR spectral matches of representative marine microdebris in juvenile coral trout. Marine microdebris, including fibres consisting of (**a**) polyester, (**b**) cotton, (**c**) polypropylene, and (**d**) keratin, were detected in the gastrointestinal tract of 20 individual juvenile coral trout (*Plectropomus leopardus* and *P. maculatus*), collected on reefs around four reef islands in the Great Barrier Reef World Heritage Area, Australia, in 2011.

Of the final 115 marine debris items detected in juvenile coral trout, 60 (52%) were classified as semi-synthetic, 48 (42%) as naturally-derived, and seven (6%) as synthetic (Table 3). These 115 items were made up of 112 fibres (97%) and three particles (3%). Items classified as synthetic consisted of four fibres and three particles, comprising of acrylic (n = 3), polyester (n = 2), polysiloxane (n = 1) and alkylphosphate ester (n = 1) (Figs 1 and 2). Primary microplastics, such as microbeads or pre-production plastic pellets, were not observed. This indicates that the microplastics detected were fragments of larger plastic items, i.e. secondary microplastics. Sixty fibres were classified as semi-synthetic, with cellulose-regenerated composites (n = 28) and natural fibre reinforced polymer composites (NFPC) (n = 22) being the most common chemical types (Figs 1 and 2). Forty-eight fibres were deemed to be naturally-derived, with cellulose (n = 27) and cellulose composites (n = 14) being the most common chemical type (Figs 1 and 2).

### Presence and abundance of marine debris in juvenile coral trout

**Figure 3:**
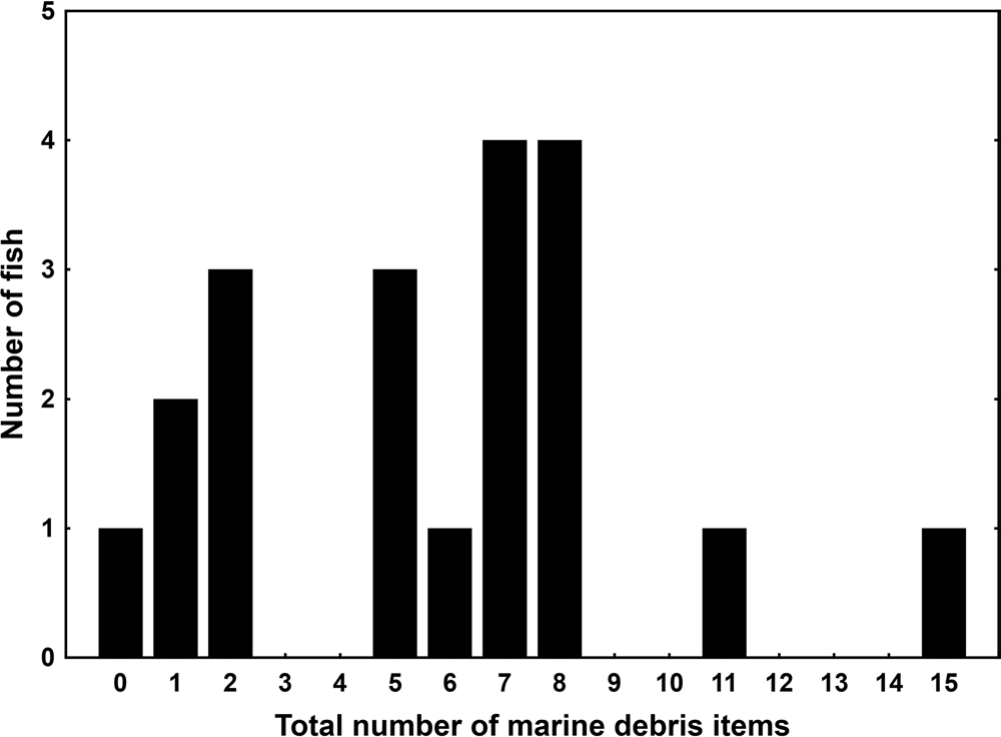
Frequency of marine microdebris items in juvenile coral trout. The number of juvenile coral trout (*Plectropomus leopardus* and *P. maculatus*) against the total number of marine microdebris items detected in the 20 individual gastrointestinal tracts. Fish were collected on reefs around four reef islands in the Great Barrier Reef World Heritage Area, Australia, in 2011.

**Figure 4:**
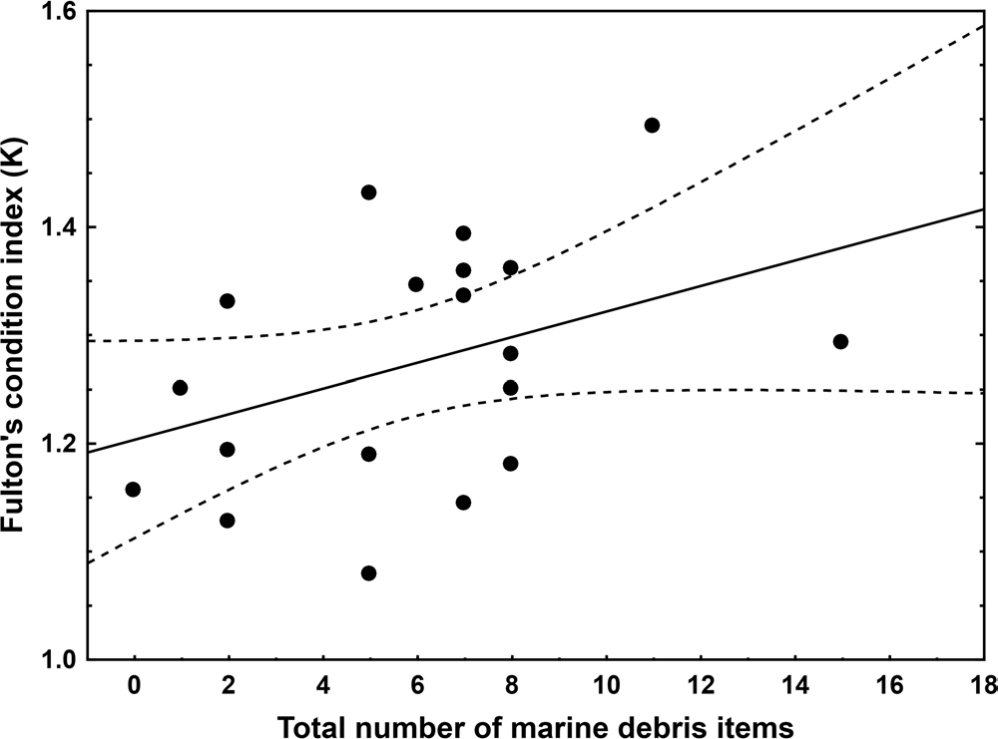
Condition of juvenile coral trout. The condition (Fulton’s condition index, K) of individual juvenile coral trout (*Plectropomus leopardus* and *P. maculatus*) plotted against the total number of marine microdebris items detected in each of the 20 gastrointestinal tract. Fish were collected on reefs around four reef islands in the Great Barrier Reef World Heritage Area, Australia, in 2011. Linear regression fit and bands (95% confidence level) are presented.

Marine debris was identified in the GIT of 19 of the 20 juvenile coral trout (Supplementary Table 1), making this the first study to report the detection of marine debris in wild-caught commercial fish on the GBR WHA. The number of marine debris items per fish ranged from zero (one fish from Lizard Island) to 15 (one fish from Heron Island), with seven and eight items being most common (Fig. 3). Across all four reef islands, the GITs contained on average 5.8 ± 0.8 standard error (s.e.m.) items per fish, and was similar across the two species examined (*P. leopardus*: 5.9 ± 1.0 s.e.m.; *P. maculatus*: 5.4 ± 1.4 s.e.m.; Supplementary Tables 1 and 2). The number of marine debris items did not change with the size of individual fish (Linear Regression: R^2^ = 0.03, F_1,18_ = 0.57, P = 0.46). The condition of individual fish, measured using Fulton’s condition index (K index), tended to increase with total number of marine debris items detected in the fish GIT (Fig. 4) albeit not significantly so (Linear Regression: R^2^ = 0.16, F_1,18_ = 3.43, P = 0.08). The most abundant colour of fibres detected was white (25%), followed by blue (13%), black (11%), and beige, white-mixed, and others (each 9%) (Table 4). Red, brown-mixed and pink fibres were the least abundant, each contributing less than 5%.

**Figure 5:**
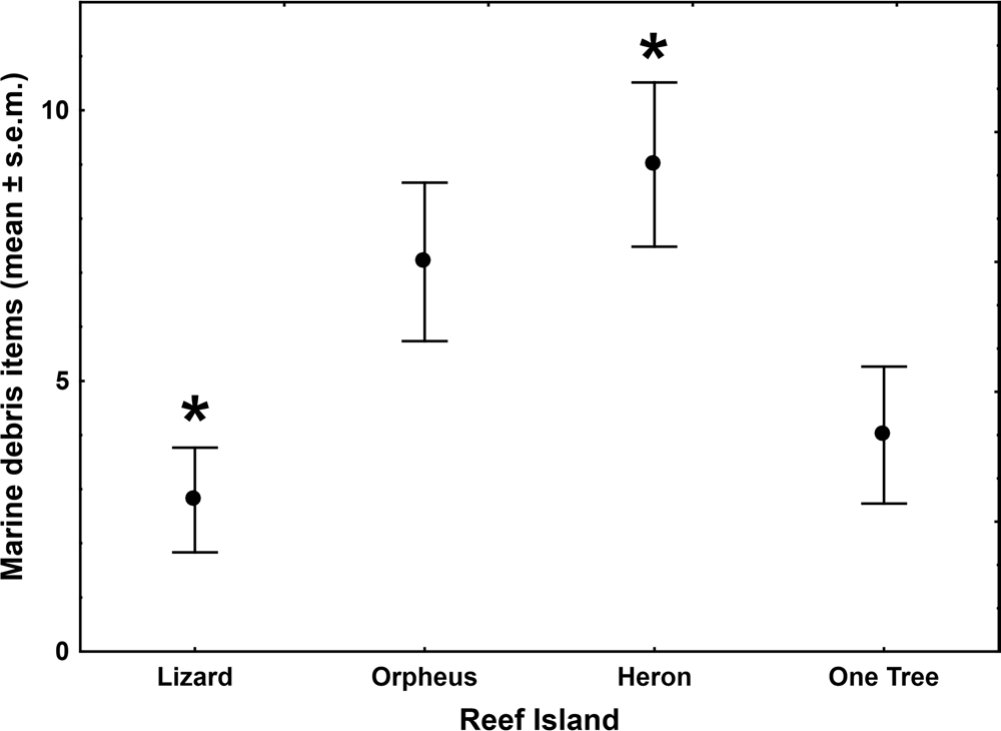
Mean number of marine microdebris items in juvenile coral trout per reef island. The average number (±s.e.m.) of marine microdebris items detected in the gastrointestinal tracts of five juvenile coral trout (*Plectropomus leopardus* and *P. maculatus*) at each of the four reef islands in the Great Barrier Reef World Heritage Area, Australia. Fish were collected in 2011.

Among the four reef islands, the mean number of marine debris items detected per fish differed significantly (One-Way ANOVA: F_3,16_ = 4.67, P = 0.016), being higher in fish collected at Heron Island compared to those from Lizard Island (Tukey HSD: P = 0.02; Fig. 5). This significant difference between the four reef islands could likely be attributed to the presence of semi-synthetic fibres (One-Way ANOVA: F_3,16_ = 3.56, P = 0.038), being higher in fish collected at Heron Island compared to those at both Lizard and Orpheus Island albeit not significantly so (Tukey HSD: P > 0.08). In contrast, the mean number of synthetic particles or fibres, or naturally-derived fibres did not differ significantly across the four islands (One-Way ANOVA: P > 0.26).

**Figure 6:**
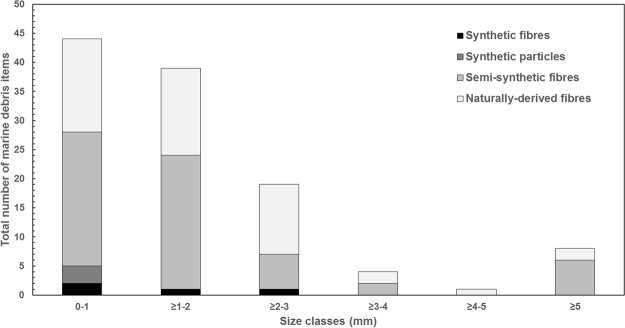
Size-frequency distribution of marine microdebris items in juvenile coral trout. Marine microdebris items were detected in the gastrointestinal tracts dissected from 19 out of the 20 individual juvenile coral trout (*Plectropomus leopardus* and *P. maculatus*), collected on reefs around four reef islands in the Great Barrier Reef World Heritage Area, Australia, in 2011.

The size distribution of the marine debris items was skewed towards the 0 to 2 mm size class, with 72% of all items being <2 mm (Fig. 6); the smallest item detected was a 0.4 mm fibre. Almost all items (93%) were between 0.4 mm and <5 mm in length and therefore considered microdebris. Only eight items (all being fibres) were ≥5 mm, with a maximum fibre length of 15.8 mm. The width of most fibres (92%) was <50 µm, and ranged from 17 µm to 72 µm. A total of 26 items (three particles, 23 fibres) between 0.4 mm and <5 mm were composed of, or contained plastic polymers (seven synthetic, 19 semi-synthetic) as determined by ATR-FTIR, and could thus be classified as microplastics. This comprises 22% of the total number of marine debris items identified in the GIT of 20 individual juvenile coral trout, and 13% of the 200 potential marine debris items first visually separated by stereomicroscopy.

## Discussion

Our review of the existing literature and the application of our proposed classification to our case study highlights the need for improved consistency in terminology for marine microdebris. Specifically, applying the classification of synthetic, semi-synthetic and naturally-derived to our case study has revealed the prevalence of ingested microdebris items that are non-plastic. This corroborates previous studies on accumulation of ingested marine debris in fish, that the majority of items can be semi-synthetic, including rayon^18,41^ and cellophane^35,44^. Notwithstanding their chemical type, such items are often incorrectly referred to as microplastics, including in the reporting of metrics on ingested marine debris^18,19,35,43^, resulting in inaccurate quantification of microplastics ingestion. In other cases, items of a determined chemical type (e.g. the semi-synthetic cellophane) are incorrectly referred to as either ‘natural’^23^ or ‘plastic’^35,43^. While recent studies have also reported marine debris of natural origin (e.g. cellulose^40^, cotton and wool^43^, keratin and chitin^44,45^), we are unaware of any studies that have subsequently examined their physical characteristics to confirm a natural or man-made origin. In our case study, only 15% (n = 29) of the 200 items visually separated as potential marine debris using stereomicroscopy were confirmed to contain plastic polymers by ATR-FTIR, with 13% (n = 26) of these 200 items being classed as microplastics (i.e. between 0.1 μm and <5 mm). These results not only suggest that most of the marine debris ingested by fish can be non-plastic in nature, but also highlight the value of our proposed classification in the accurate quantification of microplastics in environmental samples. Such information is essential to determine the impact and inform ecological risk assessments of marine debris^16,17^.

Our results demonstrate that spectroscopy is an integral part of correctly assigning chemical type to an ingested item^22,54,55^, and consequently in classifying and quantifying accumulation of ingested marine debris in fish^18,33^. Indeed, particles <2.0 mm^20^ and fibres of any size^23^ cannot be confidently classified as plastics (i.e. synthetic) using only visual examination. The first reports on ingestion of fibres in fish appeared in 2011^31,56^, however, these studies only visually examined fibres detected in fish GIT. Since then, over 30 studies have reported the presence of (micro-)fibres in fish GIT, with many of these using spectroscopy on all or a subset of the samples to establish their chemical type. These studies confirmed the ingestion of both synthetic (e.g. polyamide, polyester, polyethylene, polyethylene terephthalate, polypropylene, and polystyrene)^18,35,36,57^, and semi-synthetic (e.g. rayon, cellophane)^18,23,35^ fibres by fish. However, many of these studies using spectroscopy do not report on the specific spectral libraries used to conduct their searches against^33,35,43,57^. This makes it difficult to determine whether non-plastic semi-synthetic and naturally-derived items were searched for and not present, or were not searched for and may have been present. Stringent reporting on the libraries used to conduct searches following spectroscopy would improve the reproducibility of FTIR methods, and promote confidence in the associated results.

The application of our classification to examine the accumulation of ingested marine debris in wild-caught juvenile coral trout (*Plectropomus leopardus* and *P. maculatus*) from the GBR WHA revealed a high frequency of occurrence. Specifically, marine debris fibres and particles were found in 19 (95%) of the 20 juvenile coral trout collected from four geographically separated reef islands. Similar frequencies of occurrence (i.e. ≥80% of individuals examined) have been reported for other coastal, marine and oceanic fish species^58,59^. In juvenile coral trout, fibres comprised 97% of all the marine debris items detected, with semi-synthetic and naturally-derived fibres typifying the majority. This supports previous studies that non-plastic items can comprise a significant component of marine debris ingested by fish^18,23^, and need to be considered in assessing the potential impacts of marine microdebris. Further, these results corroborate previous studies that spectroscopic analysis is essential to establish chemical type^20,23^. This is also the first study to confirm the ingestion of naturally-derived fibres (e.g. cellulose-natural, keratin) deemed to be man-made based on their physical properties. These findings emphasize the need to systematically compare chemical (i.e. FTIR spectra) and physical (i.e. structure, shape, texture and colour) characteristics of every microdebris item to confirm chemical assignment and establish origin (i.e. natural vs man-made). The majority of fibres had a width of ≤50 µm suggesting they were derived from textiles^60^ rather than from fishing lines or ropes^57,61^. The diverse nature of synthetic, semi-synthetic and naturally-derived items detected in juvenile coral trout further highlights the need for improved and consistent terminology to classify microdebris ingestion by fish, and microdebris contamination of the marine environment in general.

The possible sources of marine debris contamination in northern Australia have been examined by several studies^62–64^. Relative to other coastal areas around Australia, the contribution of land- and sea-based sources to marine debris along the GBR coastline is unknown^63^. In GBR offshore waters and on continental islands and sand cays, both land-based sources as well as oceanic and shipping sources have been posited for the marine plastic pollution^62,64^. The source of textile fibres detected in juvenile coral trout, however, is currently unclear and could range from domestic, land-based and shipping-based sewage discharges, to (inter-)national, unknown sources that deliver fibres to the GBR WHA through oceanic or atmospheric transport. Similarly, the reason behind significantly higher numbers of marine debris items in fish from Heron Island compared to Lizard Island is unclear. This difference is unlikely to be due to sources from the islands themselves, as in 2011 all four reef islands had a research station and a resort (except One Tree Island), with associated scientific and tourism activities. Simulations using hydrodynamic numerical modelling can assist in identifying potential sources of marine debris^41,65^. Given that naturally-derived fibres tend to degrade more quickly than synthetic or semi-synthetic ones^53^, information on their relative degradation properties in tropical marine environments combined with numerical modelling, will help elucidate the potential sources of fibres detected in juvenile coral trout, and of fibres in coastal and marine waters more generally.

The accumulation of marine debris in the GIT of fish could be a result of direct ingestion from the environment, or indirect ingestion of items present in their food sources. All fish analysed in this study were from a size class that feed on benthic invertebrates and small demersal fishes^66–68^. While contamination by microplastic particles and fibres^41,64,69^ has been reported for (sub-)surface waters of the GBR WHA, no information is currently available on contamination of benthic and demersal habitats. Whether juvenile coral trout would preferentially ingest marine debris based on chemical^70^ or visual^71^ cues is currently unknown. The prevalence of white plastics in fish gut contents has been reported in some^52,72^, but not all studies^43,59^. In juvenile coral trout, the prevalence of white, as well as blue and black fibres may simply reflect their relative abundance in the GBR environment^41^. Nevertheless, selective feeding on blue microplastics that resembles natural prey (blue copepods) has been reported for Amberstripe scad *Decapterus muroadsi*^58^. The observed ingestion of marine debris could also occur via prey items; microdebris including rayon and polyester fibres were detected in the GIT of lemon damselfish (*Pomacentrus moluccensis*) collected in the central GBR WHA^41^. This species is part of the diet of *P. leopardus*^66^ with Pomacentrids being commonly taken by juveniles^67^.

The effects, detrimental or otherwise, of physical, chemical and/or microbial exposure associated with the ingestion of marine debris on wild fish populations are currently unknown. Ingestion of smaller marine debris, including microplastics has the potential to block feeding and digestive processes^73^, and can expose organisms to associated chemical contaminants^6,74^ and microorganisms^75^. In our study we did not observe any obvious signs of blockages or impaired digestion, suggesting that debris items are either digested or excreted. Digestion of the most common microfibers detected in this study, namely semi-synthetic and naturally-derived cellulosic fibres, by cellulolytic microorganisms^76,77^ is highly unlikely in carnivorous fish like *Plectropomus* species. In contrast, naturally-derived keratin fibres could be digested, e.g. by keratinases of bacteria found in biofilms^78^ and teleost fish^79,80^, given coral trout’s natural diet of benthic invertebrates and small demersal fish^66–68^. The fact that the abundance of ingested marine debris did not increase with fish size, also observed in other studies^33,59^, suggests that accumulation is ephemeral and items are excreted. Moreover, the accumulation of ingested marine debris did not appear to affect the condition of juvenile coral trout, suggesting that, at least for the fish examined, the abundance of marine debris was not detrimental to their physical condition. This is in line with findings from other studies, with only two (*Melanogrammus aeglefinus*^33^, *Girella laevifrons*^50^) out of the seventeen wild-caught fish species examined so far^33,41,45,50^ showing a decrease in condition in relation to ingested microdebris. Combined with our finding that more than 80% of over 20,000 individual fish examined did not appear to contain any marine debris, this suggests that the putative detrimental effects of marine debris ingestion on the health of wild fish populations may be overstated. To test this hypothesis, we recommend that future research apply our new level of classification and assess the probability that adverse effects will occur in wild fish populations as a result of (long-term) exposure to these different classes of marine microdebris^81^.

## Conclusion

We have developed a new classification system for marine microdebris to represent synthetic, as well as semi-synthetic and naturally-derived items. This classification system is based on a comprehensive literature review on ingestion of marine debris in coastal, marine and oceanic fish, and introduces a level of consistency in the higher-level characterization of marine microdebris. The application of this classification system to a case study on wild-caught juvenile coral trout, *Plectropomus* spp. demonstrates a high frequency of occurrence and a prevalence of semi-synthetic and naturally-derived fibres. These results highlight the importance of the use of standardised procedures and terminology for accurate characterisation and description of marine microdebris contamination. We envisage that the application of this classification system will provide improved certainty to distribution and abundance data of different chemical types, and will ultimately contribute to a more realistic assessment of potential ecological risks of marine microdebris.

## Materials and Methods

### Literature review on coastal, marine and oceanic fish

We conducted a thorough search for publications in the primary literature including the following terms: ‘marine debris’, ‘plastic’, ‘microplastic’, ‘fish’, ‘coastal’, ‘marine’, and ‘ocean*’ (i.e. all publications containing the words: ocean, oceans, oceanography, etc.). The search was performed in Web of Science™ in January 2018 and covered the years 1970 to 2017. We also reviewed several summary reports^5,82,83^ to capture information from older publications and additional reports from the grey literature. For each publication, we recorded basic information such as the species examined (including the species’ scientific name if presented in the report), the number of individuals examined, and the location of sampling.

To inform the classification and characterisation of ingested marine debris, for each report we recorded the methods used to separate and identify debris from fish samples, namely visual separation, chemical separation, photography and spectroscopy. We confirmed the presence or absence of debris particles and fibres, and noted for each whether they could be classified as synthetic, semi-synthetic or naturally-derived. Particular attention was given to the fact of whether ingested marine debris was chemically characterised using spectroscopy, as particles <2.0 mm^20^ and fibres of any size^23^ cannot be confidently classified as plastics (i.e. synthetic) using only visual examination. In such cases we noted the classification as ‘not determined’, including in cases where authors had classified these items as plastics. ‘Not determined’ was also used for particles that were visually classified as plastics but for which no size or size ranges were presented.

To inform the reporting of metrics on ingested marine debris, the findings of each publication were summarised into the number of individual fish containing marine debris and the percentage frequency of occurrence (% FO) of ingested debris (i.e. proportion of sampled individuals containing debris)^84^. More detailed information on the mean, minimum and maximum number of debris items per fish were also noted if presented in the report.

### Diet of juvenile coral trout

In our study, we focussed on juvenile coral trout (<20 cm SL) which in contrast to adults consume a considerable proportion of benthic invertebrates (Crustacea) and small demersal fishes^66–68^. This could make juveniles prone to ingesting marine debris accumulated in coral reef benthic habitats^85^. Specifically, juvenile *P. leopardus* of the ‘0’ age class (<55 mm SL) feed on benthic invertebrates and small demersal fish just above the reef slope, while the ‘1’ age class (between 100 and 200 mm SL) prey upon demersal and demersal-pelagic fishes and some invertebrates^68^. When feeding in association with other fish species, the ‘0’ age class would pick at small crustaceans and fish among the coral and sand, while the ‘1’ age class would use the school as concealment. More recent studies confirmed that small *P. leopardus* (<20 cm SL) consumed more crustaceans than larger fish^66,67^. The proportion of crustaceans decreases from almost 50% of the stomach contents for fish <10 cm SL to approximately 10% for fish between 15 and 20 cm SL^67^. Thus, the shift in diet from a large proportion of benthic invertebrates to almost complete piscivory occurs around 20 cm SL^66–68^.

### Field collections of juvenile coral trout

All methods were carried out in accordance with relevant guidelines and regulations, and all experimental protocols were approved by the GBR Marine Park Authority, the Queensland Department of Agriculture, Fisheries & Forestry, and the CSIRO Ecosystem Sciences’ Animal Ethics Committee. Juvenile common coral trout (*P. leopardus* (Lacépède)) and bar-cheek coral trout (*P. maculatus* (Bloch)) were collected around four geographically separated reef islands, namely Lizard Island, Orpheus Island, Heron Island and One Tree Island, during and immediately following the summer wet season of 2010/2011 (Fig. 1b in^86^; Table 1). The four reef islands are located within the GBR lagoon along the length of the GBR WHA, with the shortest distance from the mainland being approximately 15 km (Orpheus Island), 30 km (Lizard Island), and >60 km (Heron Island, One Tree Island). Coral trout were captured on coral rubble using either a spear gun or a fence net and anaesthetic solution (10% clove oil in seawater) in a spray bottle^87^, during April and June 2011. Following capture, fish were immediately euthanized by gill slitting and cervical dislocation, measured (total length, TL; in mm), weighed (W; in g), and dissected to preserve brain and liver tissues for ecotoxicological work unrelated to this study^86^. The remains of each individual fish, including undamaged stomach and intestines, were kept separately in resealable bags on ice until delivery to the laboratory. Remains were subsequently stored at −20 °C until GIT content analyses in June 2016.

**Table 1:** Collection information for juvenile coral trout.

Reef	Date	Distance (km)	Species	Number	Size (mm, TL)			Weight (g)		
					Mean ± s.e.m.	Min	Max	Mean ± s.e.m.	Min	Max
Lizard Island	May-11	30	*Plectopomus leopardus* (4) *P. maculatus* (1)	5	173 ± 6	157	187	63 ± 7	45	78
Orpheus Island	Apr-11 June-11	15	*Plectopomus leopardus* (1) *P. maculatus* (4)	5	182 ± 13	150	220	80 ± 14	47	120
Heron Island	May-11	65	*Plectropomus leopardus*	5	200 ± 12	157	225	107 ± 18	50	155
One Tree Island	May-11	65	*Plectropomus leopardus*	5	166 ± 25	126	238	78 ± 32	25	180

A total of 20 similarly-sized, juvenile coral trout (*Plectropomus leopardus* and *P. maculatus*) were collected around four reef islands in the Great Barrier Reef World Heritage Area, Australia, in 2011. Collection reefs and dates, distance from mainland, and number and measurements for coral trout are given. TL = total length, s.e.m. = standard error. The average size and weight of juvenile coral trout collected from the four reef islands did not differ significantly (One-way ANOVA, F_3,16_ = 0.89, P = 0.47 for average size; F_3,16_ = 0.82, P = 0.50 for average weight).

**Table 2:** Application of analysis workflow to fish gastrointestinal tract contents^20^.

Step	Process	Number of items							Reason for change in numbers
		Affected by processing step			Taken to next processing step				
		Total	Fibres	Particles	Total	Fibres	Particles	%^b^	
1	Visual separation	—	—	—	200	164	36	—	—
2	Measurement and photography	0	0	0	200	164	36	100	—
3	Chemical characterisation	11 (−5, −6)	8	3	189	156	33	95	missing, too small for ATR-FTIR; removed
4	Library interpretation of spectra	12	8	4	177	148	29	89	<60% match removed
5	Visual inspection of spectra	5	3	2	172	145	27	86	between 60 and <70% match, and very poor visual match; removed
6	Contamination check	71	68	3	—	—	—	—	≥90% match to contaminant library
7	Visual inspection of photographs	19	19	0	153	126	27	77	visual match to contaminant; removed
8	Chemical type assignment	38	14	24	115	112	3	58	natural origin could not be excluded; removed
**Number of marine microdebris items**					**115**	**112**	**3**	**58**	

The workflow is tailored to quantifying microdebris contamination, and applied to the gastrointestinal tract contents of 20 similarly-sized juvenile coral trout (*Plectropomus leopardus* and *P. maculatus*) collected on reefs around four reef islands in the Great Barrier Reef World Heritage Area, Australia, in 2011. The number of items affected by and taken to the next processing step of the analysis workflow, and the reason for a reduction in numbers is given; ^b^percentage based on total items separated using visual examination (n = 200).

**Table 3:** Chemical type assignment of marine microdebris in juvenile coral trout.

Chemical type	Fibres	Particles	Total
*SYNTHETIC*			
*Thermoplastics*			
Acrylic	1	2	3
*Thermoset and elastomer plastics*			
Polyester	2	0	2
Polysiloxane	1	0	1
*Organics*			
Alkylphosphate ester	0	1	1
*SEMI-SYNTHETIC*			
*Regenerated*			
Cellulose-regenerated	10	0	10
*Regenerated composites*			
Cellulose-regenerated: cellulose-natural	26	0	26
Cellulose-regenerated: keratin	2	0	2
*NFPCs*			
NFPC: acrylic	1	0	1
NFPC: keratin: nylon	1	0	1
NFPC: nylon	10	0	10
NFPC: polyester	3	0	3
NFPC: polypropylene	1	0	1
NFPC: polyurethane	6	0	6
*NATURALLY-DERIVED*			
*Plants*			
Cellulose	27	0	27
*Animals*			
Keratin	6	0	6
*Composites*			
Cellulose: cellulose	13	0	13
Cellulose: keratin	1	0	1
*Inorganics*			
Pigment	1	0	1
**Total**	**112**	**3**	**115**

A total of 115 marine microdebris items (112 fibres, three particles) were detected in the gastrointestinal tract of 20 similarly-sized juvenile coral trout (*Plectropomus leopardus* and *P. maculatus*) collected on reefs around four reef islands in the Great Barrier Reef World Heritage Area, Australia, in 2011. Assignment was based on the chemical type as determined by ATR-FTIR, results from Compare analyses, and visual inspection of photographs. Items were classified as synthetic, semi-synthetic, or naturally-derived. NFPC = natural fibre reinforced polymer composites.

**Table 4:** Colours of marine microdebris in juvenile coral trout.

Colour	Number	Percentage (%)
**Fibres**		
White	28	25
Blue	15	13
Black	12	11
Beige	10	9
White-mixed	10	9
Others	10	9
Blue-mixed	8	7
Brown	7	6
Red	5	4
Brown-mixed	4	4
Pink	3	3
**Total**	**112**	**100**

Number and proportion of different coloured marine microdebris items (for fibres only), detected in the gastrointestinal tract of 20 juvenile coral trout (*Plectropomus leopardus* and *P. maculatus*) collected on reefs around four reef islands in the Great Barrier Reef World Heritage Area, Australia, in 2011.

### Dissection of GIT in juvenile coral trout

The remains of 20 juvenile coral trout (15 *P. leopardus* and 5*P. maculatus*, all <20 cm SL based on the relationship between TL and SL for *P. leopardus*^88^), five from each reef island, were defrosted at room temperature and dissected at the AIMS laboratory in June 2016. Specifically, for each individual fish the entire GIT was removed from the top of the oesophagus to the rectum, and placed into an individual glass petri-dish. Dissections were conducted on a clean bench in an open laboratory rather than in a laminar flow cabinet or fume hood to minimise the possibility of marine debris in the GIT becoming airborne. To remove the GIT content, the whole GIT was cut open in the glass petri-dish and the insides exhaustively scraped clean with stainless steel straight-edged tweezers and thoroughly rinsed with Milli-Q water. The glass petri-dish containing the GIT content was covered with a glass cover to prevent loss of sample and introduction of contaminants, labelled for stereomicroscopy, and stored in a sealed container until further processing.

### Processing and analyses of marine debris in juvenile coral trout

To identify, characterise and quantify marine debris in the GIT contents, we generally followed the workflow outlined in Kroon *et al*.^20^. In brief, each GIT content sample was filtered through a 37 µm mesh. The retentate was visually inspected in a Bogorov counting chamber under a stereomicroscope (Leica M165C, 0.73x - 12.0x magnification) to identify and separate potential marine debris items. The GIT contents of each fish were thoroughly dissevered with two fine forceps and examined once. All items visually determined to have been manufactured, modified or used by people, based on absence of cellular or organic structures, shape (e.g. equal thickness), texture (e.g. patterned), and colour presence and homogeneity (e.g. blue, red, black, yellow)^16,46^ were individually selected using metal needle nose forceps or a hooked microneedle and placed onto concave glass slides. After exhaustive examination of the sample, a flat glass cover slide was placed over the concave glass slide and sealed with tape to prevent loss of items and introduction of contaminants. Items on the concave glass slide were (i) photographed at two magnifications (8x and 11x) against both black and white backgrounds (LEICA MC170 HD, LASV4.4 imaging software) to enable further visual inspection, (ii) measured to establish surface area (particles, µm^2^) or length (fibres, µm) for size distribution analysis, and (iii) measured to establish width (fibres, µm) for distinguishing between textile (≤50 µm) and other (>50 µm) sources^57,60,61^, using ImageJ (U.S. NIH, MD, USA http://rsb.info.nih.gov/ij/).

Individual items were analysed by ATR-FTIR spectroscopy as described by Kroon *et al*.^20^ and baseline corrected FTIR spectra searched against the Nicodom IR libraries (Polymers and Additives, Coatings, Fibres, Dyes and Pigments, Petrochemicals; Nicodom Ltd., Czech Republic) using Euclidian distance. A percent match (i.e. best hit) between the sample and the top reference spectrum was obtained for each individual item, and subsequently classified as low (<60%), intermediate (between 60 and <70%) and high (between ≥70 and 100%); items with a <60% match were excluded from further analyses^18,20^.

Spectra of all items with a ≥60% match were interpreted and interrogated following Workman Jr. *et al*.^54^, including a comparison of diagnostic signals in each spectrum with their best ten library matches^55^, and with spectra of all items to establish the level of similarity to each other^20^. Items with a match between 60 and <70%, no diagnostic chemical signals present, and correlations to items with a similar or lower % match and chemical signal classification, were excluded from further analysis. All items that passed spectral interpretation and interrogation were then assessed against a customised contaminant library^20^ developed specifically for this study (see below). Physical characteristics of items with percent match of ≥90% to one or more items in the contaminant library were further inspected, specifically size, shape, texture, and colour, and compared to those of the known items in the contaminant library. Items were examined conservatively, and if contamination could not be ruled out due to similarities in physical characteristics, the item was considered to have been introduced during collection, processing or analyses, and excluded from further analyses.

Initial chemical type assignment of all remaining items following the contamination check were confirmed through (i) interrogation of spectra using the PerkinElmer Compare algorithm to establish levels of similarities, and (ii) further visual inspection of photographs for physical characteristics. In addition to those physical characteristics used initially for separating potential marine debris items from the GIT^16,46^, particular attention was paid to the physical properties of fibres to establish whether they had been manufactured or modified by people. Following Norén^46^, these properties included equal thickness, no tapering towards the ends, a three-dimensional bending (i.e. not entirely straight), a clear or homogenous colouration, as well as the presence of a yarn or intertwined structure. Based on chemical type, comparison of spectra, and physical characteristics, each individual item was classified as a marine debris or natural item. Items were examined conservatively, and if a natural origin could not be ruled out the item was assigned as natural and excluded from further analyses. Marine debris items were further classified as synthetic, semi-synthetic, and naturally-derived using the definitions presented.

### Preventing contamination of juvenile coral trout samples

To prevent contamination, exposure time of samples to the surrounding environment was minimised during collection, processing and analyses, as recommended by Woodall *et al*.^89^. During field-based dissections, the dissection tray and tools were cleaned with 70% ethanol (EtOH) in Milli-Q water before use and in-between individual samples to prevent cross-contamination^86^. During the dissection and microscopic processing of the GIT contents in the AIMS laboratory, work surfaces, glassware and all dissection tools were cleaned with 70% EtOH before use and in-between individual samples^90^. While dissecting and processing the samples in the laboratory, four petri dishes filled with Milli-Q water were placed next to the work area to check for airborne contamination and later analysed as procedural blank controls. When not in direct use, all glassware and samples were covered with glass covers or aluminium foil^89,90^. The diamond compression cell of the ATR-FTIR was cleaned with methanol and lint-free tissue, and visually inspected under 2x magnification (MAGGYLAMP) between each item. Reference samples of materials used during field collection (clove oil spray bottle, fence net, resealable bags), dissection (rubber bands, nitrile gloves), and processing (lint-free tissue, laboratory coat) procedures were retained for physical characterisation, and analysed for chemical characterisation using ATR-FTIR^90^. The spectra of these reference samples were collated in a customised contaminant library developed specifically for this study.

### Data analyses

The two species of coral trout examined were considered as one single group for data analyses, given their similarities in life history, diet and feeding strategies^91^. Data analyses were conducted on items considered to be marine debris. For each individual fish, the number of debris items classified as synthetic, semi-synthetic or naturally-derived was quantified and categorised according to main chemical type. To examine potential differences in the number of marine debris items detected in juvenile coral trout among the four reef islands, a One-Way Analysis of Variance (ANOVA) with Tukey’s highest significant difference (HSD) test was used^92^. To examine the potential impact of accumulation of ingested marine debris in the GITs of juvenile coral trout, the number of these items was related to (i) the length (TL, in mm) of the fish, and (ii) the condition of individual fish, using linear regression analysis^92^. Fulton’s condition index (K index) was used to provide a measure of health for individual fish based on standard weight, using the equation ‘K = 100 (W/L^3^)’^93^, where W = weight (in gr) and L = length (TL, in mm). To examine potential preferential feeding on marine debris of particular colours, the abundance of each colour as a proportion of the total number of items detected was quantified. To determine the number and proportion of microplastics (i.e. between 0.1 μm and <5 mm) relative to non-plastic marine debris items ingested, the size frequency distributions of the three classification categories of marine debris (synthetic, semi-synthetic and naturally-derived) were plotted. Nonparametric tests were used when assumptions of normality and homogeneity of variance could not be met^92^. Tests of significance are two-tailed unless otherwise stated. Statistical analyses were conducted in Statistica^94^.

## Electronic supplementary material

Supplementary Information Text

Supplementary Dataset Table S1

## Data Availability

Data are available from the corresponding author on request.
