# Supplementary material for: Classification of marine microdebris: A review and case study on fish from the Great Barrier Reef, Australia
Source: Sci Rep. 2018 Nov 6;8:16422. doi: 10.1038/s41598-018-34590-6 (PMC6219575; doi:10.1038/s41598-018-34590-6)
Supplement: Supplementary file 1 — Supplementary Information Text [file 41598_2018_34590_MOESM1_ESM.docx]

**Classification of marine microdebris: A review and case study on fish from the Great Barrier Reef, Australia**

Frederieke J. Kroon^1,*^, Cherie E. Motti^1^, Lene H. Jensen^1^, & Kathryn L. E. Berry^1^

^1^ Australian Institute of Marine Science (AIMS), Townsville, Qld 4810, Australia

* Phone: +61 4753 4159; fax: +61 4772 5852; e-mail: [f.kroon@aims.gov.au](mailto:f.kroon@aims.gov.au).

**SUPPLEMENTARY INFORMATION**

# Supplementary Text 1.

**Aragonite**: the two particles matched with paint and pigment brands in the Nicodom library, and the results from the Compare analyses showed that they closely matched. Aragonite is the high pressure polymorph of calcium carbonate, and forms naturally in inorganic (e.g. limestone) and organic (e.g. mollusks, corals) forms. Aragonite can be white, red, yellow, orange, green, purple, grey, blue and brown. Visual inspection of the photographs of these two particles revealed that one was extremely small (<<1 mm), red and showed a pitted surface, while the other was a white, powdery chip. While the red colour was strong and appeared to be uniform, this particle was extremely small making these criteria potentially less robust. Hence, visual inspection of their photographs could not exclude a natural origin of these two particles.

**Cotton:** A total of 26 cotton fibres matched with cotton brands in the Nicodom library. Cotton is cultivated in Australia, and wild cotton (distant relatives of cultivated cottons) also occurs naturally in Australia. Visual inspection of the photographs revealed that most of these fibres (n=17) were intertwined (i.e. yarn), which does not naturally occur. In addition, most of these fibres (n=15) had non-natural cotton colours such as blue, red, brown, black, white-red, white-blue, brown-blue, and blue-red. Hence, a total of 24 cotton fibres were classified as naturally-derived. The natural origin of two cotton fibres, however, could not be excluded, based on their colour (white), shape (uneven width), and structure (not intertwined).

**Keratin:** A total of 37 fibres and particles matched with commercial and animal keratin products (such as feather, fur, hair, leather, and wool) in the Nicodom library. Keratin polymers are all basically the same, and the Nicodom library cannot distinguish against different animals. The results from the Compare analyses of these fibres and particles showed close similarity, confirming their chemical type assignment to keratin. Visual inspection of the photographs revealed that some of these fibres (n=4) were intertwined (i.e. yarn), which does not naturally occur. In addition the width of some of these fibres (n=4) was uniform, which suggests they have been made or modified by humans. Hence, a total of 6 keratin fibres were classified as naturally-derived. In contrast, a natural origin could not be excluded for the remaining keratin fibres (n=12) and particles (n=19) based on their physical characteristics such as colour (transparent, white, gold, beige, brown, dark brown), coloration (not uniform), shape (flake, sliver), and structure (irregular).

**Pigments:** A total of 2 pigment items matched with pigments in the Nicodom library. The pigment fibre matched to henna red, which is made from lawsone. Careful examination of the spectrum showed polymer signals indicative of lawsone. Even though lawsone is natural, fibres are not naturally dyed with lawsone. Furthermore, this fibre had a consistent width indicating it has been manipulated. Hence, this fibre was assigned as naturally-derived. The pigment particle matched with Bohemian Green Earth, and its colour (beige) and shape (sliver) could not rule out a natural origin.

**Rayon**: A total of 10 fibres matched with rayon in the Nicodom library. Rayon is derived synthetically from cellulose fibre, and while made from natural polymers, it does not naturally occur. Their manufactured origin was confirmed by visually inspecting the photographs, showing intertwined fibres (n=3), and non-natural coloration such as blue, red, white-blue, and blue-orange (n=4).

**Silicate**: A total of 2 particles matched with a natural pigment and commercial paint in the Nicodom library. Calcium silicate is formed in lime-soil systems under thermal conditions, and it is this property that has enabled its manufacture from natural sources for use as cement. Visual inspection of the photographs showed a flake and a round-ish particle, respectively, with both items of a beige colour. Based on these physical characteristics a natural origin could not be ruled out.

**Sisal:** One sisal fibre matched natural sisal in the Nicodom library. Sisal is a species of Agave native to southern Mexico; its fibres are used in making various products such as rope, twine, paper, cloth, footwear, hats, bags, carpets, and dartboards. This long fibre had a consistent width and as such was classified as naturally-derived.

**Straw:** Two straw fibres matched natural straw in the Nicodom library. Based on the intertwined structure of one of the fibres, and the colours of both fibres (blue and red), these fibres were classified as naturally-derived.

# Supplementary **Table S1.**

See Excel file.

# Supplementary **Table S2.**

Total number of marine microdebris items, classified as synthetic, semi-synthetic and naturally-derived, detected in the gastrointestinal tract of 20 similarly-sized, juvenile coral trout (*Plectropomus leopardus* and *P. maculatus*) collected on reefs around four reef islands in the Great Barrier Reef World Heritage Area, Australia, in 2011. Assignment was based on the chemical type as determined by ATR-FTIR, results from Compare analyses, and visual inspection of photographs.

| **Reef** | **Fish** | | **Number of marine microdebris items** | | | | |
| --- | --- | --- | --- | --- | --- | --- | --- |
|  |  |  | **Synthetic** | | **Semi-synthetic** | **Naturally-derived** | **Total** |
|  | **Species** | **Number** | **Fibres** | **Particles** | **Fibres** | **Fibres** |  |
| Lizard Island | *P. leopardus* | 1 | 0 | 0 | 4 | 1 | 5 |
|  | *P. leopardus* | 2 | 0 | 0 | 2 | 3 | 5 |
|  | *P. leopardus* | 3 | 0 | 0 | 1 | 1 | 2 |
|  | *P. leopardus* | 4 | 0 | 0 | 0 | 0 | 0 |
|  | *P. maculatus* | 5 | 0 | 0 | 1 | 1 | 2 |
| Orpheus Island | *P. maculatus* | 1 | 0 | 0 | 5 | 2 | 7 |
|  | *P. maculatus* | 2 | 0 | 0 | 6 | 2 | 8 |
|  | *P. leopardus* | 3 | 2 | 0 | 3 | 6 | 11 |
|  | *P. maculatus* | 4 | 0 | 0 | 5 | 3 | 8 |
|  | *P. maculatus* | 5 | 0 | 0 | 1 | 1 | 2 |
| Heron Island | *P. leopardus* | 1 | 0 | 2 | 8 | 5 | 15 |
|  | *P. leopardus* | 2 | 1 | 0 | 6 | 1 | 8 |
|  | *P. leopardus* | 3 | 1 | 0 | 3 | 3 | 7 |
|  | *P. leopardus* | 4 | 0 | 0 | 1 | 6 | 7 |
|  | *P. leopardus* | 5 | 0 | 0 | 6 | 2 | 8 |
| One Tree Island | *P. leopardus* | 1 | 0 | 0 | 1 | 0 | 1 |
|  | *P. leopardus* | 2 | 0 | 0 | 0 | 1 | 1 |
|  | *P. leopardus* | 3 | 0 | 1 | 2 | 2 | 5 |
|  | *P. leopardus* | 4 | 0 | 0 | 3 | 3 | 6 |
|  | *P. leopardus* | 5 | 0 | 0 | 2 | 5 | 7 |
| **Total** |  | **20** | **4** | **3** | **60** | **48** | **115** |

# References (Supplementary Table S1 in Excel)

1. Kubota, T. & Uyeno, T. Food habits of Lancetfish *Alepisaurus ferox* (Order Myctophiformes) in Suruga Bay, Japan. *Jap. J. Ichthyol.* **17**, 22-28 (1970).
2. Carpenter, E. J., Anderson, S. J., Harvey, G. R., Miklas, H. P. & Peck, B. B. Polystyrene spherules in coastal waters. *Science* **178**, 749-750 (1972).
3. Kartar, S., Milne, R. A. & Sainsbury, M. Polystyrene waste in the Severn Estuary. *Mar. Pollut. Bull.* **4**, 144 (1973).
4. Manooch III, C. S. Food habits of yearling and adult Striped bass, *Morone saxatilis* (Walbaum), from Albemarle Sound, North Carolina. *Chesapeake Sci.* **14**, 73-86 (1973).
5. Colton Jr., J. B., Knapp, F. D. & Burns, B. R. Plastic particles in surface waters of the Northwestern Atlantic. *Science* **185**, 491-497 (1974).
6. Anon. Plastic cups found in fish. *Mar. Pollut. Bull.* **6**, 148 (1975).
7. Kartar, S., Abou-Seedo, F. & Sainsbury, M. Polystyrene spherules in the Severn Estuary — A progress report. *Mar. Pollut. Bull.* **7**, 52 (1976).
8. Manooch, I. I. I. C. S. & Hogarth, W. T. Stomach contents and giant trematodes from Wahoo, *Acanthocybium Solanderi*, collected along the South Atlantic and Gulf coasts of the United States. *Bull. Mar. Sci.* **33**, 227-238 (1983).
9. Manooch III, C. S., Mason, D. L. & Nelson, R. S. Foods of Little tunny *Euthynnus alletteratus* collected along the Southeastern and Gulf Coasts of the United States. *Nippon Suisan Gakk.* **51**, 1207-1218 (1985).
10. Hoss, D. E. & Settle, L. R. Ingestion of plastics by teleost fish. In *Proceedings of the Second International Conference on Marine Debris.* (eds R.S. Shomura & H.L. Codfrey) 693-709 (US Department of Commerce, NOAA Technical Memorandum NMFS, 1990).
11. Laist, D. W. Impacts of marine debris: entanglement of marine life in marine debris including a comprehensive list of species with entanglement and ingestion records. In *Marine debris. Sources, Impacts, and Solutions* *Springer Series on Environmental Management* (eds J.M. Coe & D.B. Rogers) 99-139 (Springer, 1997).
12. Kubota, T. Synthetic materials found in the stomachs of Longnose lancetfish collected from Suruga Bay, central Japan. In *Second International Conference on Marine Debris, 1989.* (eds R.S. Shomura & M.L. Godfrey) 710-717 (NOAA-TM-NMFS-SWFSC-154, 1990).
13. Jackson, G. D., Buxton, N. G. & George, M. J. A. Diet of the Southern opah *Lampris immaculatus* on the Patagonian Shelf; the significance of the squid *Moroteuthis ingens* and anthropogenic plastic. *Mar. Ecol. Prog. Ser.* **206**, 261-271 (2000).
14. Fujieda, S., Uchiyama, M., Azuma, T., Fukuda, R. & Arita, Y. Ingestion case of plastics by Black marlin *Makaira indica* and Lancetfish *Alepisaurus ferox* caught in the East Indian Ocean. *Mem. Fac. Fish. Kagoshima Univ.* **57**, 47-48 (2008).
15. Boerger, C. M., Lattin, G. L., Moore, S. L. & Moore, C. J. Plastic ingestion by planktivorous fishes in the North Pacific central gyre. *Mar. Pollut. Bull.* **60**, 2275-2278 (2010).
16. Davison, P. & Asch, R. G. Plastic ingestion by mesopelagic fishes in the North Pacific subtropical gyre. *Mar. Ecol. Prog. Ser.* **432**, 173-180 (2011).
17. Possatto, F. E., Barletta, M., Costa, M. F., do Sul, J. A. & Dantas, D. V. Plastic debris ingestion by marine catfish: an unexpected fisheries impact. *Mar. Pollut. Bull.* **62**, 1098-1102 (2011).
18. Dantas, D. V., Barletta, M. & da Costa, M. F. The seasonal and spatial patterns of ingestion of polyfilament nylon fragments by estuarine drums (Sciaenidae). *Environ.* Sci. *Pollut. Res.* **19**, 600-606 (2012).
19. Ramos, J. A. A., Barletta, M. & Costa, M. F. Ingestion of nylon threads by Gerreidae while using a tropical estuary as foraging grounds. *Aquatic Biol.* **17**, 29-34 (2012).
20. Anastasopoulou, A., Mytilineou, C., Smith, C. J. & Papadopoulou, K. N. Plastic debris ingested by deep-water fish of the Ionian Sea (Eastern Mediterranean). *Deep-Sea Res. Part I-Oceanogr. Res. Pap.* **74**, 11-13 (2013).
21. Choy, C. A. & Drazen, J. C. Plastic for dinner? Observations of frequent debris ingestion by pelagic predatory fishes from the central North Pacific. *Mar. Ecol. Prog. Ser.* **485**, 155-163 (2013).
22. Foekema, E. M. *et al.* Plastic in North Sea fish. *Environ. Sci. Technol.* **47**, 8818-8824 (2013).
23. Jantz, L. A., Morishige, C. L., Bruland, G. L. & Lepczyk, C. A. Ingestion of plastic marine debris by longnose lancetfish (*Alepisaurus ferox*) in the North Pacific Ocean. *Mar. Pollut. Bull.* **69**, 97-104 (2013).
24. Lusher, A. L., McHugh, M. & Thompson, R. C. Occurrence of microplastics in the gastrointestinal tract of pelagic and demersal fish from the English Channel. *Mar. Pollut. Bull.* **67**, 94-99 (2013).
25. Madeira Di Beneditto, A. P. & Awabdi, D. R. How marine debris ingestion differs among megafauna species in a tropical coastal area. *Mar. Pollut. Bull.* **88**, 86-90 (2014).
26. Avio, C. G., Gorbi, S. & Regoli, F. Experimental development of a new protocol for extraction and characterization of microplastics in fish tissues: First observations in commercial species from Adriatic Sea. *Mar. Environ. Res.* **111**, 18-26 (2015).
27. Collard, F., Gilbert, B., Eppe, G., Parmentier, E. & Das, K. Detection of anthropogenic particles in fish stomachs: an isolation method adapted to identification by Raman Spectroscopy. *Arch. Environ. Contam. Toxicol.* **69**, 331-339 (2015).
28. Neves, D., Sobral, P., Ferreira, J. L. & Pereira, T. Microplastics ingestion by commercial fish off the Portuguese coast. *Mar. Pollut. Bull.* **101**, 119-126 (2015).
29. Phillips, M. B. & Bonner, T. H. Occurrence and amount of microplastic ingested by fishes in watersheds of the Gulf of Mexico. *Mar. Pollut. Bull.* **100**, 264-269(2015).
30. Rochman, C. M. *et al.* Anthropogenic debris in seafood: Plastic debris and fibers from textiles in fish and bivalves sold for human consumption. *Sci. Rep.* **5**, 14340 (2015).
31. Romeo, T. *et al.* First evidence of presence of plastic debris in stomach of large pelagic fish in the Mediterranean Sea. *Mar. Pollut. Bull.* **95**, 358-361 (2015).
32. Battaglia, P. *et al.* Diet and first documented data on plastic ingestion of *Trachinotus ovatus* L. 1758 (Pisces: Carangidae) from the Strait of Messina (central Mediterranean Sea). *Ital. J. Zool.* **83**, 121-129 (2016).
33. Bellas, J., Martinez-Armental, J., Martinez-Camara, A., Besada, V. & Martinez-Gomez, C. Ingestion of microplastics by demersal fish from the Spanish Atlantic and Mediterranean coasts. *Mar. Pollut. Bull.* **109**, 55-60 (2016).
34. Bråte, I. L. N., Eidsvoll, D. P., Steindal, C. C. & Thomas, K. V. Plastic ingestion by Atlantic cod (*Gadus morhua*) from the Norwegian coast. *Mar. Pollut. Bull.* **112**, 105-110 (2016).
35. Cannon, S. M. E., Lavers, J. L. & Figueiredo, B. Plastic ingestion by fish in the Southern Hemisphere: A baseline study and review of methods. *Mar. Pollut. Bull.* **107**, 286-291 (2016).
36. Ferreira, G. V. B. *et al.* Plastic debris contamination in the life cycle of Acoupa weakfish (*Cynoscion acoupa*) in a tropical estuary. *ICES J. Mar. Sci.* **73**, 2695-2707 (2016).
37. Liboiron, M. *et al.* Low plastic ingestion rate in Atlantic cod (*Gadus morhua*) from Newfoundland destined for human consumption collected through citizen science methods. *Mar. Pollut. Bull.* **113**, 428-437 (2016).
38. Lenz, R., Enders, K., Beer, S., Sørensen, T. K. & Stedmo, S. A. Analysis of microplastic in the stomachs of herring and cod from the North Sea and Baltic Sea. 30 (DTU Aqua, National Institute of Aquatic Resources, Denmark) (2016).
39. Lusher, A. L., O'Donnell, C., Officer, R. & O'Connor, I. Microplastic interactions with North Atlantic mesopelagic fish. *ICES J. Mar. Sci.* **73**, 1214-1225 (2016).
40. Miranda, D. d. A. & de Carvalho-Souza, G. F. Are we eating plastic-ingesting fish? *Mar. Pollut. Bull.* **103** (2016).
41. Nadal, M. A., Alomar, C. & Deudero, S. High levels of microplastic ingestion by the semipelagic fish bogue *Boops boops* (L.) around the Balearic Islands. *Environ. Pollut.* **214**, 517-523 (2016).
42. Naidoo, T., Smit, A. J. & Glassom, D. Plastic ingestion by estuarine mullet *Mugil cephalus* (Mugilidae) in an urban harbour, KwaZulu-Natal, South Africa. *Afr. J. Mar. Sci.* **38**, 145-149 (2016).
43. Romeo, T., Peda, C., Fossi, M. C., Andaloro, F. & Battaglia, P. First record of plastic debris in the stomach of Mediterranean lanternfishes. *Acta Adriat.* **57**, 115-123 (2016).
44. Rummel, C. D. *et al.* Plastic ingestion by pelagic and demersal fish from the North Sea and Baltic Sea. *Mar. Pollut. Bull.* **102**, 134-141 (2016).
45. Tanaka, K. & Takada, H. Microplastic fragments and microbeads in digestive tracts of planktivorous fish from urban coastal waters. *Sci. Rep.* **6**, 34351 (2016).
46. Wesch, C., Barthel, A.-K., Braun, U., Klein, R. & Paulus, M. No microplastics in benthic eelpout (*Zoarces viviparus*): An urgent need for spectroscopic analyses in microplastic detection. *Environ. Res.* **148**, 36-38 (2016).
47. Alomar, C. & Deudero, S. Evidence of microplastic ingestion in the shark *Galeus melastomus* Rafinesque, 1810 in the continental shelf off the western Mediterranean Sea. *Environ. Pollut.* **223**, 223-229 (2017).
48. Alomar, C. *et al.* Microplastic ingestion by *Mullus surmuletus* Linnaeus, 1758 fish and its potential for causing oxidative stress. *Environ. Res.* **159**, 135-142 (2017).
49. Avio, C. G., Cardelli, L. R., Gorbi, S., Pellegrini, D. & Regoli, F. Microplastics pollution after the removal of the Costa Concordia wreck: First evidences from a biomonitoring case study. *Environ. Pollut.* **227**, 207-214 (2017).
50. Collard, F. *et al.* Microplastics in livers of European anchovies (*Engraulis encrasicolus*, L.). *Environ. Pollut.* **229**, 1000-1005 (2017).
51. Collard, F. *et al.* Morphology of the filtration apparatus of three planktivorous fishes and relation with ingested anthropogenic particles. *Mar. Pollut. Bull.* **116**, 182-191 (2017).
52. Guven, O., Gokdag, K., Jovanovic, B. & Kideys, A. E. Microplastic litter composition of the Turkish territorial waters of the Mediterranean Sea, and its occurrence in the gastrointestinal tract of fish. *Environ. Pollut.* **223**, 286-294 (2017).
53. Hermsen, E., Pompe, R., Besseling, E. & Koelmans, A. A. Detection of low numbers of microplastics in North Sea fish using strict quality assurance criteria. *Mar. Pollut. Bull.* **122**, 253-258 (2017).
54. Jabeen, K. *et al.* Microplastics and mesoplastics in fish from coastal and fresh waters of China. *Environ. Pollut.* **221**, 141-149 (2017).
55. Jensen, L. H., Ingestion of microplastic by *Pomacentrus moluccensis* and the occurrence of microplastics in the surface water of the Great Barrier Reef. MSc Thesis. 62 (Department of Biology, University of Copenhagen, Copenhagen, Denmark, 2017).
56. Karlsson, T. M. *et al.* Screening for microplastics in sediment, water, marine invertebrates and fish: Method development and microplastic accumulation. *Mar. Pollut. Bull.* **122**, 403-408 (2017).
57. McGoran, A. R., Clark, P. F. & Morritt, D. Presence of microplastic in the digestive tracts of European flounder, *Platichthys flesus*, and European smelt, *Osmerus eperlanus*, from the River Thames. *Environ. Pollut.* **220**, 744-751 (2017).
58. Mizraji, R. *et al.* Is the feeding type related with the content of microplastics in intertidal fish gut? *Mar. Pollut. Bull.* **116**, 498-500 (2017).
59. Murphy, F., Russell, M., Ewins, C. & Quinn, B. The uptake of macroplastic & microplastic by demersal & pelagic fish in the Northeast Atlantic around Scotland. *Mar. Pollut. Bull.* **122**, 353-359 (2017).
60. Naidoo, T., Goordiyal, K. & Glassom, D. Are nitric acid (HNO3) digestions efficient in isolating microplastics from juvenile fish? *Water, Air, Soil Pollut.* **228**, 470 (2017).
61. Ory, N. L., Sobral, P., Ferreira, J. L. & Thiel, M. Amberstripe scad *Decapterus muroadsi* (Carangidae) fish ingest blue microplastics resembling their copepod prey along the coast of Rapa Nui (Easter Island) in the South Pacific subtropical gyre. *Sci. Total Environ.* **586**, 430-437 (2017).
62. Peters, C. A., Thomas, P. A., Rieper, K. B. & Bratton, S. P. Foraging preferences influence microplastic ingestion by six marine fish species from the Texas Gulf coast. *Mar. Pollut. Bull.* **124**, 82-88 (2017).
63. Steer, M., Cole, M., Thompson, R. C. & Lindeque, P. K. Microplastic ingestion in fish larvae in the western English Channel. *Environ. Pollut.* **226**, 250-259 (2017).
64. Sun, X. *et al.* Ingestion of microplastics by natural zooplankton groups in the northern South China Sea. *Mar. Pollut. Bull.* **115**, 217-224 (2017).
65. Vendel, A. L. *et al.* Widespread microplastic ingestion by fish assemblages in tropical estuaries subjected to anthropogenic pressures. *Mar. Pollut. Bull.* **117**, 448-455 (2017).
66. Wagner, J. *et al.* Novel method for the extraction and identification of microplastics in ocean trawl and fish gut matrices. *Anal. Methods* **9**, 1479-1490 (2017).
